# Enhancing Intelligence: From the Group to the Individual

**DOI:** 10.3390/jintelligence6010011

**Published:** 2018-03-01

**Authors:** Roberto Colom, Francisco J. Román

**Affiliations:** Departamento de Psicología Biológica y Salud, Facultad de Psicología, Universidad Autónoma de Madrid, 28049 Madrid, Spain; fcojavier.roman.psi@gmail.com

**Keywords:** intelligence enhancement, cognitive training, brain structure

## Abstract

Research aimed at testing whether short-term training programs can enhance intelligence is mainly concentrated on behavior. Expected positive effects are found sometimes, but the evidence is far from conclusive. It is assumed that training must evoke changes in the brain for observing genuine improvements in behavior. However, behavioral and brain data are seldom combined in the same study. Here we present one example of this latter type of research summarizing, discussing, and integrating already published results. The training program was based on the adaptive dual n-back task, and participants completed a comprehensive battery measuring fluid and crystallized ability, along with working memory and attention control, before and after training. They were also submitted to MRI scanning at baseline and post-training. Behavioral results revealed positive effects for visuospatial processing across cognitive domains. Brain imaging data were analyzed by longitudinal voxel-based morphometry, tensor-based morphometry, surface-based morphometry, and structural connectivity. The integration of these multimodal brain results provides clues about those observed in behavior. Our findings, along with previous research and current technological advances, are considered from the perspective that we now live in ideal times for (a) moving from the group to the individual and (b) developing personalized training programs.

## 1. Introduction

Here we summarize, discuss and integrate several previously published research papers based on behavioral and neuroimaging data registered from the same sample [1,2,3,4,5]. The integrative effort leads to the goal of enhancing intelligence through intervention programs that increase their efficiency by applying a perspective based on the individual rather than on the group. Illustrative novel descriptive analyses, along with some theoretical perspectives and available research findings, are consistent with the perspective endorsed here.

First, we provide clues regarding the intelligence construct, since our training program was aimed at increasing intelligent performance as assessed by standardized tests. Accumulated evidence—such as the Flynn effect—demonstrates that intelligence can be improved [6,7], but only if specific requirements can be met [8,9,10,11]. The integration of behavioral and brain imaging data should help us to increase our understanding regarding the responsiveness to targeted cognitive intervention programs. We subscribe to the view that cognitive challenges must be individualized for maximizing successful outcomes [12].

Second, we summarize, discuss and integrate behavioral/cognitive findings from a research program designed to increase fluid reasoning ability and working memory capacity using an intervention program based on a challenging working memory task [13]. Novel individualized results will be presented for illustrative purposes and for supporting our perspective.

Third, brain-imaging findings obtained from the same sample will be summarized, discussed and integrated. These imaging results are consistent with the behavioral/cognitive findings: the training group outperformed controls in visuospatial processing skills cutting across cognitive domains [1]. This key observation will be related to models of human cognitive abilities based on content (verbal/spatial) rather than process (working memory, executive control). Although this was unexpected, the content-based approach might be better than the process-based approach for making sense of the behavioral-brain findings integrated here.

Fourth, we will discuss how the individualized perspective can be based on available research findings and recent technological developments. The crucial questions concern how individuals’ brain networks are uniquely organized and how this organization relates to behavior. Again, and for illustrative purposes only, we will provide some new neuroimaging results obtained from individuals selected from our group. Results show unique cortical changes from pretest to posttest in MRI registrations: individuals can show high or low cognitive training improvements, and these same individuals can experience high or low changes in fluid reasoning (Gf).

Finally, we discuss evidence favoring the individualized approach. This is the overall message: individual profiles must be specified before designing the intervention best fitted for enhancing the ability of interest, mainly because individual sensitivity to exogenous perturbations might be critical both for brain processes and for the related cognitive abilities.

## 2. Behavior, Cognition, and the Brain

General intelligence (*g*) is an integrative cognitive ability comprised of a broad array of >80 narrower abilities, such as fluid reasoning, crystallized knowledge and skills, and visuospatial processing ability [14]. These abilities are hierarchically organized, *g* captures the variance shared by them all, and there is substantial performance variance unexplained by the higher-order abilities [15,16]. Scientists discuss the interpretation of the positive manifold giving rise to the structure of human intelligence [14,17,18,19], but the relevant point here is that intelligence is a complex human trait responsive to the environment, as indisputably demonstrated by the Flynn effect.

James Flynn has proposed one comprehensive framework for integrating three levels of analysis regarding intelligence research: BIDS (B = Brain, ID = Individual differences, S = Social trends). The level shown by real world cognitive skills changes over time (social trends). Individual differences in intelligence are substantially correlated (within times) because there are (presumably) underlying latent traits such as general cognitive ability (*g*). Finally, there are both general (e.g., blood supply) and specific (e.g., neural regions) factors in the brain relevant for intelligence. Brain research “*should show the beneficial effects of cognitive exercise throughout life*” [6]. In this regard, the brain might be thought of as a muscle because of its susceptibility to change (plasticity). Challenging cognitive tasks require the involvement of the brain at global and regional levels, and different challenges would recruit distinguishable regions in the brain, along with their structural and functional connections [7].

Indeed, it is acknowledged that mental function causes changes in brain structure and function in humans [20] and non-human animals [21]. However, the mechanisms behind the observed structural and functional variations in the brain that are associated with cognitive changes in response to some type of intervention are still mysterious. Challenging cognitive tasks might lead to increased resting-state spontaneous activity in neural networks, which could be useful for preparing the brain to provide finer reactions in the near future [22], but it is unclear if cognitive changes require brain changes or if the latter would inevitably lead to the former.

Lövdén et al. [23] proposed one comprehensive framework for the study of cognitive plasticity based on the following key points: (1) cognitive and brain functions are adaptive and variable; (2) plasticity requires structural brain changes with functional outcomes; (3) interventions may evoke plastic changes if they are based on a mismatch between supply (capacity of the system) and (environmental) demands; (4) the supply-demand mismatch is present when demands are greater than the available supply or when the supply is greater than the demands; (5) triggering plastic changes requires a supply-demand mismatch; and (6) individual differences in supply-demand mismatch and observed plasticity levels are correlated. In short, substantive plastic changes will be more likely when the (cognitive) system is systematically challenged using the appropriate (intervention) demands. From here, we can predict that challenges should be personalized for maximizing successful outcomes. 

Earl B. Hunt [24] and Richard J. Haier [12] share the view that understanding human intelligence differences requires increasing our comprehension of how the brain works: “*intelligence is 100% a biological phenomenon, genetic or not, influenced by the environment or not, and the relevant biology takes place in the brain*” [12]. This perspective has been translated into ‘The Brain Connection’ (TBC) perspective, meaning that understanding the processes supporting the integration of the genotype and the environment, taking place in the brain, will provide the proper framework for moving forward [17]. Therefore, we must find ways for integrating behavioral/cognitive and brain data, accepting that we do not know in advance if they will work systematically in tandem.

Haier [12] goes one step further claiming that scientists devoted to the study of human intelligence differences are mainly interested in developing methods for enhancing this psychological factor in the entire population. All efforts made for studying intellectual differences, regarding their structure (factor models), their function (cognitive models) and the related brain structures and functions (biological models) are ultimately oriented towards this goal. Consistent with this perspective, Ralph Adolphs [25] raises this key problem in neuroscience: “*how could science make everybody’s brain function best?*”.

In short, scientists agree in that finding methods for enhancing intelligence deserves close and sustained attention. There is a long tradition in psychology and related fields oriented towards achieving this general goal, but the fact is that there are high expectations but the evidence is still arguable [26,27,28]. Here we will summarize, discuss and integrate behavioral/cognitive and brain findings derived from research designed for increasing fluid reasoning ability using an intervention based on a challenging adaptive task tapping working memory related processes. We will conclude by suggesting a change of perspective that may eventually lead to novel insights and fruitful outcomes.

## 3. Cognition and Brain Networks: Summary, Discussion, and Integration of Previously Published Findings

Here we summarize a research program that tested whether a challenging and adaptive cognitive intervention (a dual n-back task) (a) has any impact on structural brain features and (b) can increase general fluid reasoning ability and working memory capacity beyond the expected spontaneous improvements by the mere effect of practice. We begin by providing details regarding the general framework, and, afterwards, we discuss and integrate findings observed at behavioral/cognitive and brain levels.

### 3.1. Framework

**Figure 1:**
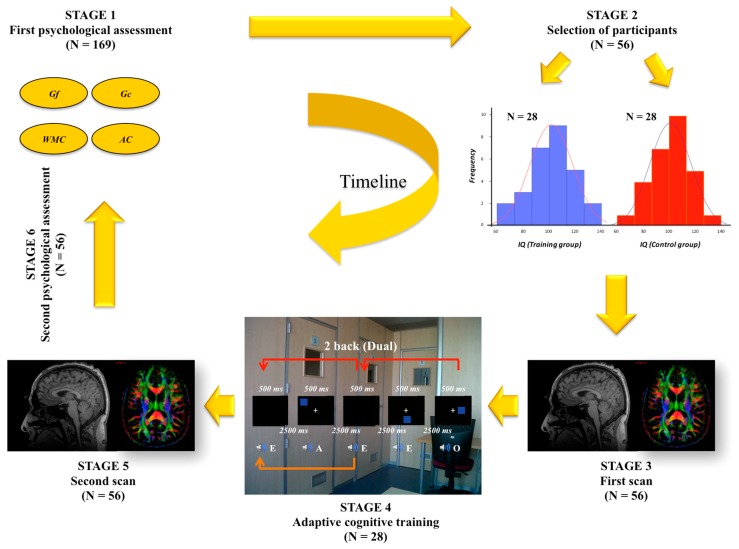
Timeline of the research program described in the present article.

One straightforward test-retest design was the main framework for our research program. Figure 1 depicts the timeline including first psychological assessment (stage 1), selection/recruitment of participants (stage 2), first MRI scan (stage 3), adaptive cognitive training (stage 4), second MRI scan (stage 5), and second psychological assessment (stage 6).

169 university undergraduates completed a comprehensive psychological test battery tapping fluid reasoning (Gf), crystallized ability (Gc), working memory capacity (WMC), and attention control (AC). As recommended [29,30,31], each of these psychological factors was assessed by three measures. Gf was measured with the Raven Advanced Progressive Matrices (RAPM), the abstract reasoning subtest from the Differential Aptitude Test (DAT-AR), and the inductive reasoning subtest from the Primary Mental Abilities Battery (PMA-R). Gc was measured with the verbal reasoning subtest from the DAT (DAT-VR), the numerical reasoning subtest from the DAT (DAT-NR), and the vocabulary subtest from the PMA (PMA-V). WMC was measured with the reading span (verbal), the computation span (numerical), and the dot matrix (spatial) tasks. Finally, AC was measured with cognitive tasks based on the quick management of conflict: verbal (vowel–consonant) and numerical (odd–even) flanker tasks, along with the spatial (right–left) Simon task. A detailed description of these measures can be found in Colom et al. [1]. Cronbach’s alpha values (corrected by the Spearman–Brown prediction formula) for all intelligence tests were appropriate (Table 1). Therefore, the measurement model included four factors and twelve measures. The latent factors showed correlations ranging from 0.33 (Gf-AC) to 0.98 (Gf-Gc) in the sample of 169 individuals (the correlation between Gf and Gc in the participants finally recruited was of 0.63, *p* < 0.001).

Fifty-six women were recruited according to their performance on measures tapping Gf and Gc. Half were assigned to the training group and the other half were submitted to the passive control group. Both groups were carefully matched, and the widest possible range of scores on cognitive ability was represented in them. We note that this number of participants is appropriate for addressing statistical power issues in brain imaging analyses [32]. We also note that Au et al.’s [33] meta-analysis showed that Hawthorne artifacts are irrelevant for the type of training considered here: active controls fail to outperform passive controls. There is “*no evidence that the effects of n-back training can be explained by Hawthorne effects*”.

The recruited participants were scanned for obtaining structural data of their brains (3D T1 and DTI), along with resting state functional MRIs—only structural findings will be discussed. Once done this first registration wave, the training group completed the adaptive cognitive program, whose specific details are detailed elsewhere [1]. After 24 training sessions (twelve weeks, 2 sessions per week) both groups were scanned again using the same imaging protocols and they also completed the second psychological assessment tapping the same cognitive factors.

The completion of these six stages allowed the accumulation of behavioral/cognitive and brain data required for finding answers to the research questions noted above. Technical details and exhaustive results regarding behavior/cognition can be found in Colom et al. [1], whereas those related with the brain are reported in Colom et al. [2,3] and Román et al. [4,5]. Here we briefly discuss and integrate the key findings leading to a general picture of the evidence and its probable consequences for orienting future research aimed at enhancing intelligence.

### 3.2. Behavior/Cognition

The main assumption behind the intervention program was that the trained cognitive processes would have measurable impact on people’s fluid intelligence and working memory capacity. Completing the adaptive dual n-back task requires working memory related processes such as encoding, maintenance, and retrieval [34]. As discussed by Martinez et al. [35] ‘encoding’ requires converting perceptual information into mental representations, ‘maintenance’ requires preserving the relevant information in the short-term—avoiding decay by time passing and interference—and ‘retrieval’ allows recovering the relevant information when required.

Working memory and fluid reasoning share common capacity limitations based on (a) the number of items that can be reliably kept active in the short-term (memory span) and (b) the number of relationships among elements active during the reasoning flow [17,36,37,38]. These shared limitations might rely on the ability to build and keep relevant connections in the short-term for coping with concurrent cognitive challenges. Therefore, overcoming memory span limitations should impact fluid reasoning ability even when both cognitive factors do not share superficial similarities.

**Figure 2:**
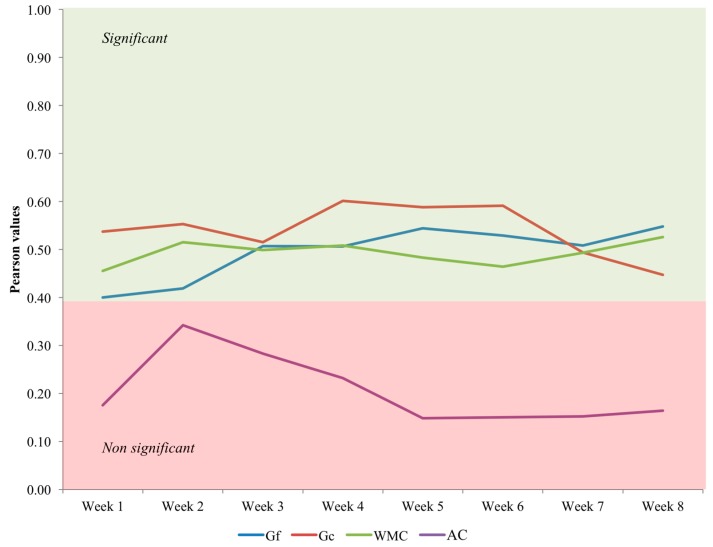
Correlations between baseline cognitive levels (Gf = fluid reasoning ability, Gc = crystallized ability, WMC = working memory capacity, AC = attention control) and adaptive dual n-back performance across weeks. The first four weeks were devoted to visual single n-back and auditory single n-back training.

**Figure 3:**
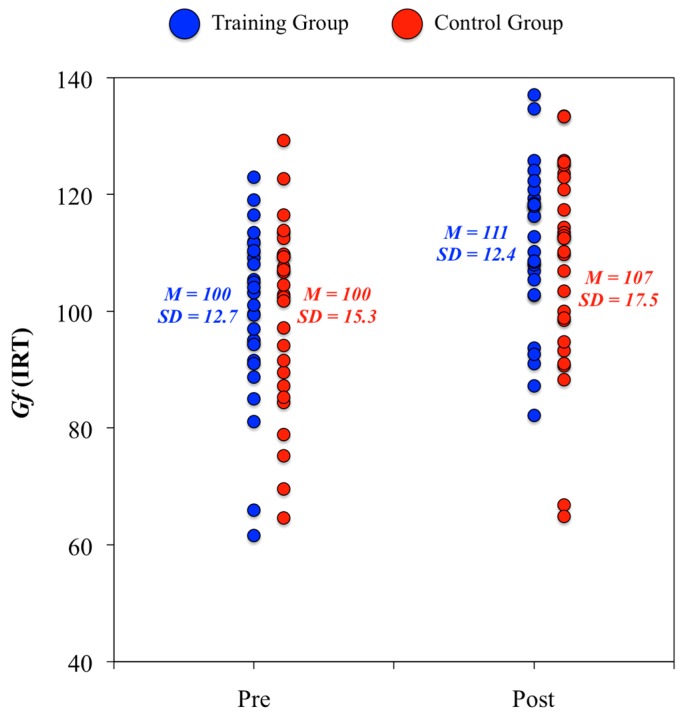
Individual score changes in fluid ability from the pretest to the posttest psychological evaluation.

**Figure 4:**
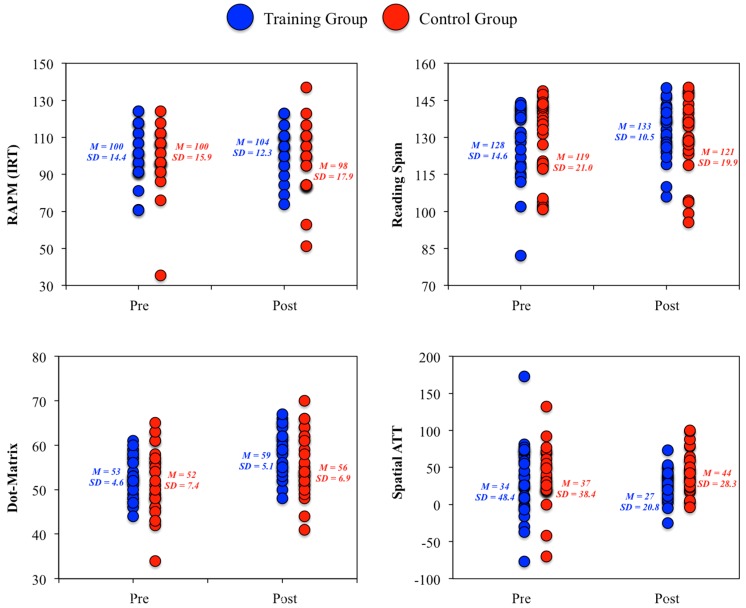
Individual changes in the RAPM (Raven Advanced Progressive Matrices) test, the Reading span working memory task, the Dot Matrix working memory task, and the Spatial attention control task.

The behavioral/cognitive results led to several conclusions:

1. There are statistically significant correlations between n-back performance differences across training sessions and fluid reasoning ability/crystallized ability/working memory capacity differences assessed at baseline. Therefore, individuals showing higher scores on these three psychological factors from the outset are those presenting better training performance. These results demonstrate that the intervention precludes automatization and remains challenging across sessions (Figure 2).

2. At the construct level, we did not find any statistically significant difference between groups from the pretest to the posttest in their psychological evaluations (ANCOVA analyses: DV = score at posttest, IV = group, and Covariate = score at pretest). The standardized change in Gf for the training group was equivalent to eleven IQ points (*d* = 0.75), whereas this value for the control group was equivalent to seven IQ points (*d* = 0.51) (Figure 3). There were null pretest-posttest changes in Gc for both groups. Furthermore, it is worth noting that the test-retest correlation in Gf was above 0.80 for the training (*r* = 0.81) and control (*r* = 0.84) groups, and, therefore, participants showing higher pretest scores were those showing better posttest scores.

3. As underscored by Colom et al. [1] constructs are not homogeneous. Therefore, results at the measures level were also analyzed, again with ANCOVA models, and this led to an interesting revelation: statistically significant group differences favoring the training group were observed for the RAPM test, along with Reading Span, Dot Matrix, and Spatial Attention Control tasks (Figure 4). All these measures require visuospatial processing skills. Therefore, tests and tasks tapping this type of content across cognitive domains (fluid reasoning, working memory, and attention control) were sensitive to training. This finding was consistent with Melby-Lervåg and Hulme’s [39] meta-analysis and Stephenson and Halpern’s [40] results. In the latter study, significant Gf improvements were observed after intensive training in visuospatial short-term memory tasks.

Cognitive performance can be analyzed at different levels of analysis. Scores can be summarized by latent constructs, but these do not exhaust individuals’ performance measures. The findings showed a lack of statistically significant group differences at the construct level, but analyses at the measures level revealed one consistent pattern for measures tapping visuospatial processing across cognitive domains.

*Novel evidence at the individual level*. There were large individual differences in performance changes observed across the adaptive dual n-back training program, as well as in fluid reasoning ability (Gf), from pretest to posttest sessions. Specifically, dual n-back changes ranged from 1 to 7 (mean change = 2.8, SD = 1.6), whereas Gf changes ranged from −7 to +30 (mean change = 10.4, SD = 8.3).

Individuals showing low n-back changes (<2, N = 10), average n-back changes (2–4, N = 11), and high n-back changes (>4, N = 5) showed closely similar Gf changes (11.5, 9.4, and 10.4 points, respectively). However, within n-back groups large individual differences were observed: those with low n-back changes showed Gf changes ranging from −3 to +30, those with average n-back changes showed Gf changes ranging from −7 to +21, and those with high n-back changes showed Gf changes ranging from 0 to +18. The lack of any pattern is summarized by the null correlation between n-back changes across the training program and fluid reasoning changes from pretest to posttest assessments (Pearson *r* = −0.09, Spearman Rho = −0.03). There were changes in Gf ranging from −7 to +30, but they did not have any relationship with n-back improvements across training sessions.

### 3.3. The Brain

Behavior/Cognitive findings provide a complex picture. The anticipated positive impact of the training program over fluid reasoning ability (far transfer) and working memory capacity (near transfer), at the construct level, was absent. Nevertheless, tests and tasks characterized by their visuospatial processing requirements were sensitive to training.

Our research framework allows examination of brain features to determine whether there are networks related to the measured cognitive differences, both between and within groups. We now discuss and integrate results derived from four neuroimaging approaches: longitudinal voxel-based morphometry (VBM), tensor-based morphometry (TBM), surface-based morphometry (SBM), and structural connectivity (SC). Specific technical details associated with these approaches are detailed elsewhere [2,3,4,5].

**Figure 5:**
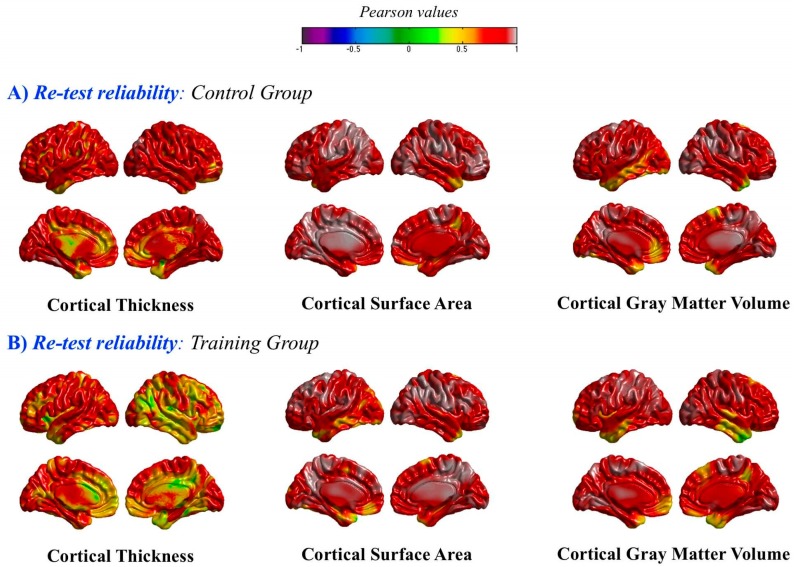
Pre-post Pearson correlations at the vertex level (**A**) in the control group and (**B**) in the training group, for the three surface-based morphometry (SBM) indices: thickness, surface area, and volume.

As noted above, both groups were matched according to their Gf/Gc scores. They were also equivalent in their brain indices at pretest. Furthermore, reliability values for these indices were satisfactory. Thus, for instance, the pre-post correlation for gray matter volumes estimated with SPM was 0.92 for the training group and 0.96 for controls. The computed pre-post correlation—at the vertex level—for SBM measures (thickness, surface area, and volume) are depicted in Figure 5. Again, values were very high for both groups across the brain (> 0.80). Nevertheless, reliability values were a bit lower for cortical thickness in the training group for some regions of the right hemisphere.

To follow our discussion the reader needs a small number of tips.

*Neuroimaging analyses*. All the computed neuroimaging analyses were based on group × time interactions, as recommended [40], and these were the main questions: (a) Are there differential structural changes in brain for the training group as compared with the matched controls? (b) Are these changes related to performance differences in the training program? (c) Are standardized changes in a set of psychological factors (fluid and crystallized ability, working memory capacity, and attention control) measured before and after training, related to structural brain changes? 

VBM has been the most popular approach for testing gray matter changes following a given intervention [41,42]. Longitudinal VBM allows measuring brain changes over time considering the characteristics of intra-subject analysis. TBM has never been applied for studying the effects of cognitive short-term training programs aimed at enhancing working memory related processes in healthy individuals [3]. TBM builds 3D maps of volumetric changes (Jacobian determinants) by matching brain scans acquired at two time-points, and, therefore, it is appropriate for the automated mapping of brain changes across time.

Nevertheless, brain volumes combine independent cortical measures, namely, thickness (CT) and surface area (CSA) [43,44]. SBM approaches allow us to obtain proper measures of these two cortical features. The number of radial columns perpendicular to the pial surface defines CSA, whereas the horizontal layers in the cortical columns define CT. CSA represents functional units in the brain, whereas CT has been related to cortical connectivity.

Finally, we relied on the structural connectome framework using network-based statistics (NBS) and graph theoretical analyses. Diffusion-weighted images were analyzed for anatomical connectome reconstruction and structural connectivity changes and NBS group comparisons across time were computed. The training and control groups were compared using different graph-theory indices (clustering, characteristic path length, global and local efficiency, and strength) for all identified connections and for the sub-networks derived from NBS analyses. Finally, correlations between these graph indices and (a) training performance and (b) standardized changes for the cognitive domains (measured before and after training) were computed.

**Figure 6:**
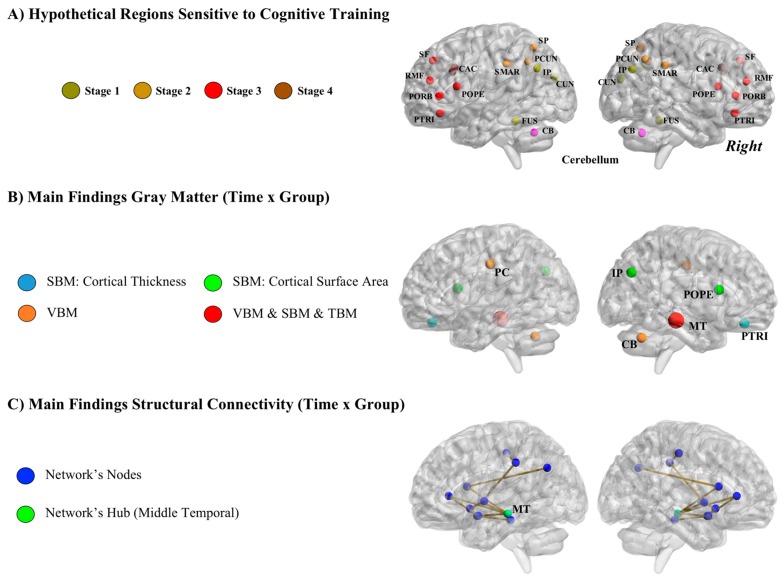
(**A**) Hypothetical regions sensitive to training defined after the parietal-frontal integration theory of intelligence (P-FIT). Stage 1, CUN = Cuneus, FUS = Fusiform Gyrus, IP = Inferior Parietal; Stage 2, PCUN = Precuneus, SP = Superior Parietal, SMAR = Supramarginal Gyrus; Stage 3 = POPE = Pars Opercularis, PORB = Pars Orbitalis, PTRI = Pars Triangularis, RMF = Rostral Middle Frontal, SF = Superior Frontal; Stage 4, CAC = Caudal Anterior Cingulate. (**B**) Summary of findings derived from longitudinal VBM (voxel-based morphometry), TBM (tensor-based morphometry), and SBM (surface-based morphometry) analyses. IP = Inferior Parietal, MT = Middle Temporal, POPE = Pars Opercularis, PTRI = Pars Triangularis, PC = Posterior Cingulate, CB = Cerebellum. (**C**) Summary of findings derived from structural connectivity analyses.

Figure 6 depicts (a) the network of brain regions that should be sensitive to the completed training according to both theory and previous research (Figure 6A); (b) findings derived from VBM, TBM, and SBM analyses (Figure 6B); and (c) findings derived from structural connectivity (SC) analyses (Figure 6C).

The adaptive dual n-back task completed by the training group requires (a) monitoring simultaneous visual and auditory incoming information to properly update the working memory system; (b) switching between these two types of information for getting proper answers when required; and (c) applying inhibitory processes to avoid making errors of omission and commission along the continuous flow of incoming information [34].

Therefore, we expected differential structural brain changes after training mainly in the parietal-frontal network [45,46] comprising regions relevant for on-line short-term storage and cognitive control, along with changes in the cerebellum (connected with frontal and parietal regions) because the cognitive training program requires executive updating and skill acquisition (Figure 6A).

Furthermore, because of the shared capacity limitations between fluid reasoning ability and working memory capacity highlighted above, we expected associations between observed training-related brain changes and standardized changes in fluid reasoning ability and working memory capacity assessed before and after training. This association was not hypothesized for crystallized ability and attention control.

Finally, we predicted increased structural connectivity within brain regions underlying fluid reasoning ability and working memory capacity, because the training regime engages common processes. Therefore, we expected frontal and parietal involvement in the sub-network detected by NBS. We also predicted increased global efficiency (Eg), local efficiency (El) and strength (S) for the sub-network sensitive to training, because of the expected increased inter-regional communication. This sub-network should show better small worldness properties after cognitive training. Therefore, increased clustering (Cl) and decreased characteristic path length (L) were expected.

Relevant findings are discussed and integrated in the next section.

#### 3.3.1. VBM, TBM and SBM

*Longitudinal VBM*. The results showed statistically significant group differences in gray matter clusters of voxels within the left posterior cingulate cortex, right cerebellum, and right temporal lobe (Figure 6B). Gray matter volume in these clusters was greater in the training group in the pretest-posttest comparison. These regions are connected with the parietal-frontal network and are thought to support cognitive processes relevant for the completed training regime: selection of the relevant information, inhibition of irrelevant and distracting stimuli, switching between competing requirements (processing of visual and auditory information), keeping track of dual incoming information, and selecting the proper response. Nevertheless, specific frontal-parietal regions were not identified (Figure 6A).

The second relevant finding was that gray matter changes in these three regions were not significantly related with performance across training sessions. We identified, however, negative correlations between the measured cognitive and brain indices, namely, n-back performance gains and pretest-posttest gray matter changes: the greater the gray matter changes, the smaller the cognitive gains across the training program. We will discuss this potentially relevant issue below.

The third result revealed null correlations between pretest-posttest changes in the four psychological factors (fluid reasoning, crystallized ability, working memory capacity, and attention control) and gray matter volume changes. Therefore, the latter failed to relate to any consistent pattern regarding the observed cognitive changes in the near and far transfer psychological factors (working memory capacity and fluid reasoning, respectively). This null finding might be attributed to the combination of independent cortical features, namely, thickness and surface area.

*TBM*. The comparison of the Jacobian determinants obtained from the training and control groups revealed greater changes in the former group in the right cerebellum, bilateral temporal, bilateral prefrontal, and bilateral inferior parietal regions, but only the temporal region survived the correction for multiple comparisons. The subsequent analyses focused on this latter region. 

A statistically significant negative correlation was found between mean brain changes and dual n-back training performance: individuals showing greater expansions improved less across cognitive training sessions. Finally, null values were found when the Jacobian determinants (brain changes) were correlated with pretest-posttest changes in the four psychological factors assessed before and after training. This result was consistent with the observed after computing longitudinal VBM.

Negative correlations between brain changes and adaptive n-back performance were obtained after applying VBM and TBM. VBM results failed to reach statistical significance, but a negative correlation was consistently observed for the three gray matter clusters. TBM results led to a large and statistically significant correlation. These findings suggest that individuals highly successful in the training program do have the required processing resources, whereas those individuals less successful do not. The effortful processing required and sustained across twelve weeks might be behind their greater structural brain changes. Indeed, individuals with large n-back training improvements showed better cognitive scores from the outset, and, therefore, the required processing would be least challenging for them. On the other hand, low achievers showed lower cognitive scores at baseline, and, therefore, their brains might be more sensitive to sustained effort. A lack of processing resources may be behind the greater changes observed in their brain structures in response to the systematic cognitive challenge. In this regard, animal studies have revealed that effort involved in learning is necessary for evoking structural brain changes [47].

*SBM*. This third neuroimaging method added additional information relevant to understanding the effects of training. Images obtained in the scanner were divided into twelve regions of interest (ROIs), for both thickness and surface area, defined by brain areas distinguished by their genetic substrate [43,44]. Following the basic research framework shared across methods, the training and control groups were compared across time (group × time interactions). Regions showing statistically significant group differences were in the middle temporal lobe, ventral frontal, parietal cortices, and (frontal) pars opercularis (Figure 6B). All these regions are known to support working memory related processes.

The training group showed cortical preservation with respect to the control group in ventral frontal thickness, right (frontal) pars opercularis surface area, and in the right middle temporal lobe for both cortical indices. Statistically significant differences were mainly located in temporal and frontal regions. Interestingly, despite their lack of spatial contiguity, these regions share genetic influences. Furthermore, all the statistically significant brain changes were in the right hemisphere. As discussed below, this may be consistent with the behavioral/cognitive results observed at the measures level [1].

Afterwards, we compared the pattern observed at the brain level with cognitive differences at baseline. The cortical thinning observed in the control group was circumscribed to those individuals showing lower intelligence scores at baseline. Individuals in the training group showed remarkable cortical preservation irrespective of individuals’ baseline intelligence levels. This led to the suggestion that training compensates for low baseline intelligence: practicing a challenging cognitive task might prevent spontaneous dendritic and spine elimination. Therefore, (a) gray matter preservation was equivalent for high intelligence individuals in both the training and control groups; but (b) only low intelligence individuals from the training group showed the noted preservation.

There are several studies addressing the effects of short-term interventions over structural brain indices. Thus, for instance, Ilg et al. [48] investigated the effects of a 2-week practice period of mirror reading. They combined structural and functional brain data in order to investigate overlaps among regions. Deactivations were observed in the right superior parietal cortex, along with increased activation in the right dorsal occipital cortex. The latter result was associated with increased gray matter volume in this same area. Haier, Karama, Leyba and Jung [49] found thickening in frontal and temporal areas after playing an online version of Tetris over three months (1.5 h per week on average). In a very short cognitive intervention (3 days) carried out by Kwok et al. [50] reliable structural gray matter changes were observed. Mackey, Whitaker and Bunge [51] focused on changes in white matter after 3 months of reasoning training with law students, finding decrements in radial diffusivity and mean diffusivity. Therefore, the practice period considered in our research is appropriate for finding reliable structural changes in the brain in response to cognitive training.

#### 3.3.2. Structural Connectivity

The computed group × time interactions showed enhanced connectivity for the training group in a network composed of brain regions supporting the cognitive processes required by the training program [52] (Figure 6C). The node showing the greatest increase in connectivity values was located in the middle temporal region. This area might be a central hub for communication across regions included in the sub-network identified. Of note is that the gray matter analyses summarized above for this sample identified this same brain region as sensitive to changes after the completed training.

Analyses of the identified nodes revealed their role for the following cognitive functions: (a) middle temporal > interference resolution; (b) pars triangularis, pars opercularis, and pallidum > inhibitory control; (c) parietal (supramarginal, inferior parietal, and postcentral), parahippocampal, and insula > working memory and intelligence; (d) accumbens and forebrain > engagement. Therefore, beyond nodes relevant for higher-order cognition (intelligence and working memory) we detected further nodes related to inhibitory processes and engagement with the trained task.

Regarding changes in connectome topological properties, the time × group interaction was statistically significant for global efficiency (Eg) and strength (S). Specifically, the training group showed enhanced Eg and S after training, while controls remained at the same levels observed at baseline. Networks with high Eg levels are more efficient [46]. S is related with the degree in a weighted network and this degree reflects the number of nodes to which a given node is connected (in a binary network). However, connectional changes failed to show significant correlations with both dual n-back performance levels across the training program and posttest-pretest changes in the four psychological factors assessed before and after training. Therefore, structural connectivity changes were not associated with the psychological covariates.

### 3.4. Integration and Conclusions

Structural results obtained after applying longitudinal VBM, TBM, and SBM methods for comparing groups (training versus control) across time (before versus after training) underscore the following brain regions (Figure 6B): right orbitofrontal (thickness), right pars opercularis (surface area), right inferior parietal (surface area), right middle temporal (volume, surface, thickness), right cerebellum (volume), and left posterior cingulate (volume). Most regions are located in the right hemisphere and they belong to frontal, parietal, and temporal regions. (Frontal) pars opercularis, inferior parietal and middle temporal nodes/regions were also identified in the structural connectome analyses (Figure 6C). Therefore, this temporal-parietal-frontal network seems to be sensitive to cognitive intervention: those individuals completing the training showed greater brain changes within this network than the matched controls.

We chose these brain regions sensitive to the intervention for answering two relevant questions focusing on the training group:

(1) Are brain changes related to cognitive performance across training sessions?
(2) Are brain changes related to changes in near (working memory capacity) and far (fluid reasoning ability) transfer psychological factors measured before and after training?

Regarding the second question, we failed to find significant relationships: observed psychological and brain changes were dissociated at the group level. With respect to the first question, we found negative relationships between brain changes and training performance: individuals showing smaller dual n-back gains across sessions were those showing greater brain changes. Lower cognitive ability levels at baseline characterized these individuals, and therefore, we reasoned that the challenging training sustained across three months was behind their brain changes. On the other hand, higher cognitive ability levels at baseline characterized individuals with larger n-back gains, and, therefore, they had the required mental resources for copying successfully with the challenging sustained training (no brain changes required).

In this regard, SBM analyses revealed that brighter individuals from the control group showed brain changes equivalent to those observed for individuals of the training group (irrespective of their cognitive levels at baseline). Therefore, the training program seems to attenuate spontaneous cortical thinning processes identified for individuals showing low cognitive ability at baseline. This led us to suggest that (a) baseline cognitive ability does have some substantive role over both training performance levels and cortical changes; (b) facing one challenging cognitive requirement modifies expected cortical changes; and (c) high ability individuals may seek spontaneously cognitive challenges impacting over their cortical development. This latter point might be related with the requirement to demonstrate that changes after training do have some impact on real-life settings [53,54].

On the other hand, the concentration of brain changes observed in the right hemisphere was interpreted as being consistent with the behavioral/cognitive results. Remember that, at the measures level, the training group outperformed controls in tests and tasks tapping visuospatial processing skills cutting across cognitive domains (fluid reasoning, working memory, and attention control).

In this latter regard, McFarland [55] published a review article answering the next question: how can neuroscience inform the study of individual differences in cognitive abilities? After discussing several lines of evidence, he arrived at the conclusion that: “*brain networks might best be characterized in terms of the information they process rather than in terms of abstract psychological processes such as working memory and executive control*”. Models of human cognitive abilities use latent constructs showing substantial conceptual overlap [19,56], and, therefore, these models might be best characterized by their contents rather than their processes, resulting in the classification of cognitive abilities according to a hierarchy of content.

This content-based approach is consistent with both modern psychometric models and older cognitive approaches. The VPR (Verbal-Perceptual-Rotation) model [57] distinguishes between high-order verbal and non-verbal cognitive factors on the intelligence hierarchy: “*the model is consistent with the idea of coordination of function across brain regions and with the known importance of brain laterality in intellectual performance*”. The left hemisphere is more involved in verbal processes, whereas the right hemisphere controls non-verbal/spatially-oriented processes. 

On the other hand, more than forty years ago, Paivio [58] proposed the dual-coding theory (DCT) of human cognition (see also [59]). In a more recent review article, Paivio made an explicit connection among intelligence (psychometric models), cognition (DCT), and the brain [60]. He [60] raises reservations about the P-FIT model [45] because, in his view, it fails to address the empirical evidence summarized by the DCT regarding verbal and nonverbal brain correlates. This theory underscores referential synergistic interconnections between the verbal and non-verbal mind. Referential associations between these two types of information “*permit objects to be named and names to activate images that represent world knowledge*”.

In short, our results showing impact of the training program over visuospatial processing skills tapped by tests and tasks across cognitive domains might be coherent with this content-based approach. We began with a process-based approach, but we end with a change of perspective after integrating the key findings. In addition, as underscored by Johnson and Bouchard [57], neuronal growth is a process that takes place within an individual and understanding why cognitive performance “*takes the particular pattern it does requires comparison across individuals*”. After studying the structural brain correlates of verbal and non-verbal dimensions orthogonal to the general factor of intelligence (*g*), Johnson et al. [61] concluded: the same test or task may not measure the same abilities in the same ways in different individuals. This strongly suggests we must move from the group to the individual, as discussed in the final section.

## 4. The Future: From the Group to the Individual

Dubois and Adolphs [62] highlighted the relevance of pursuing a “*fully personalized investigation of brain function*”. Current research efforts are shifting from group comparisons to the analysis of individual differences in brain function. We must pay attention to these differences for improving our understanding of crucial psychological (intelligence, personality) and socio-demographic factors (age, sex, culture).

Dubois and Adolphs propose the following general pipeline for this individualized research approach:

1. Acquire raw data in the scanner applying minimal preprocessing (realignment, field inhomogeneity correction, grand mean scaling, and so forth).
2. Warp volumetrically subcortical areas and register the cortex to a surface template using multimodal surface matching.
3. Select whole-brain or ROIs analyses and project data into a common space.
4. Model preprocessed data and compute multidimensional statistics for an individual.
5. Use individual statistics for predicting individual measures, such as general cognitive ability level or group classification (e.g., training versus control).
6. Compare full and null models to obtain the unique predictive value of the imaging statistic.

Moving from the group to the individual is now feasible thanks to recent technological advances such as automated cortical surface reconstruction, connectivity informatics, multimodal surface matching, or hyperalignment, to name some of them.

The study by Finn et al. [63] is a good example of this individualized approach. Their report begins with this acknowledged, but frequently neglected, statement: “*all individuals are unique*”. Brain structure and function show huge individual differences, but group difference studies set aside within-group heterogeneity. Their functional connectome analyses showed that individualized profiles “*act as an identifying fingerprint*”, meaning that these profiles uniquely identify distinct individuals. Furthermore, the obtained functional connectivity profiles predicted individual differences in fluid reasoning ability. This research concluded that neuroimaging studies done with humans must shift from group-level inferences to inferences about individual subjects. The relevant question is how individuals’ networks are uniquely organized and how this organization relates to behavior/cognition.

**Figure 7:**
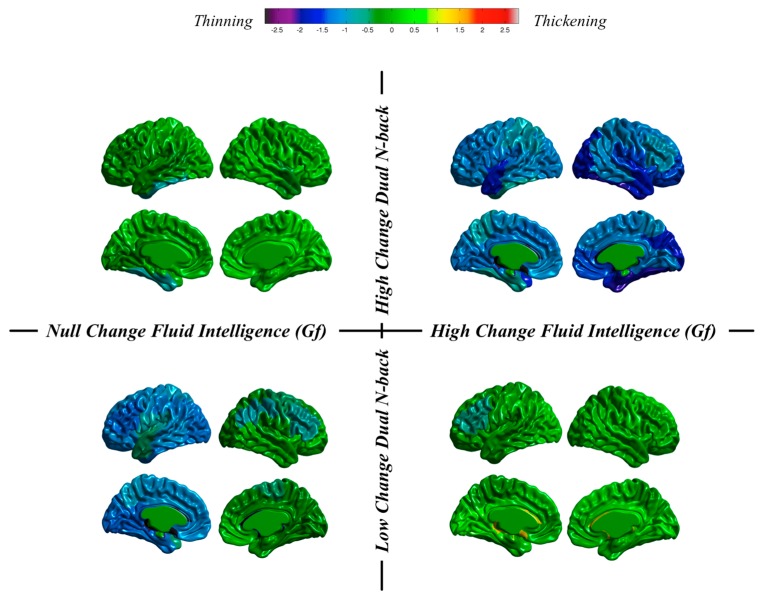
Thickness changes for individuals showing null changes in fluid ability and high changes in dual n-back (**top left**), high changes in fluid ability and high changes in dual n-back (**top right**), null changes in fluid ability and low changes in dual n-back (**bottom left**), and high changes in fluid ability and low changes in dual n-back (**bottom right**).

**Figure 8:**
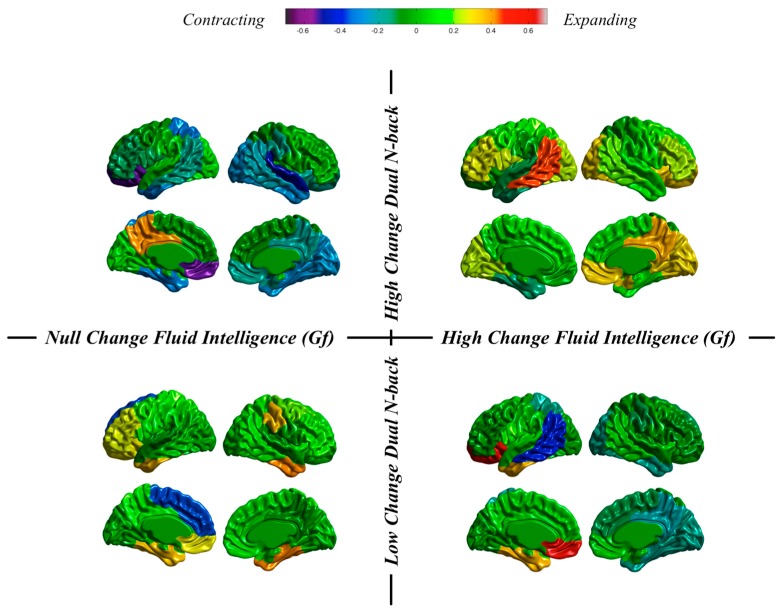
Surface area changes for individuals showing null changes in fluid ability and high changes in dual n-back (**top left**), high changes in fluid ability and high changes in dual n-back (**top right**), null changes in fluid ability and low changes in dual n-back (**bottom left**), and high changes in fluid ability and low changes in dual n-back (**bottom right**).

*Individuals and groups revisited*. We can take a close look at results obtained at the individual level in our dataset. We do that by now focusing on changes from pretest to the posttest sessions in thickness and surface area. These changes will be inspected after individuals showing high or low training changes and high or low changes in fluid reasoning (Figure 7 and Figure 8).

Regarding thickness (Figure 7), we can see (a) preservation for an individual showing high dual n-back changes and null changes in Gf, as well as for an individual showing low n-back changes and high changes in Gf; and (b) thinning for individuals showing low or high n-back changes and null or high changes in Gf.

Results for surface area (Figure 8) show (a) contraction, preservation and expansion for individuals showing low n-back changes and null or high changes in Gf; (b) contraction, preservation, and expansion for an individual showing high n-back changes and null changes in Gf; and (c) preservation and expansion for an individual showing high n-back changes and high changes in Gf.

These are highly individualized, or unique, cortical changes from the pretest to the posttest MRI registrations: (a) almost null dual n-back gains across the entire training regime can be accompanied by null or high changes in Gf along with thinning or thickness preservation; and (b) high dual n-back improvements across the training program can be accompanied by null or high changes in Gf along with thickness preservation or generalized thinning. Therefore, the main conclusion is this: individuals can show high or low cognitive training changes, but these same individuals can experience high or low changes in Gf. Furthermore, different combinations are unrelated with changes observed in brain structure.

“*All individuals are unique*”.

## 5. Conclusions: Personalized Training

Clark et al.’s [64] study provides one example of how integrating psychological and biological data can address the issue of what is meant by cognitive enhancement. They determined brain regions supporting the identification of objects embedded in a complex virtual reality environment and examined brain changes across one unguided learning process. Afterwards, brain stimulation was applied for increasing learning rate. Functional MRI and transcranial direct-current stimulation (tDCS) were combined for finding the brain networks supporting the learning process. As a result of the guided brain stimulation, participants required less time for achieving targeted learning outcomes. They concluded that tDCS guided by neuroimaging results can benefit training effectiveness.

Santarnecchi and Rossi [65] suggested that intelligence is strongly related with ‘system-level brain plasticity’. In this regard, Santarnecchi et al. [66] have shown that individuals with high intelligence levels preserve system efficiency when their brain networks are attacked. The simultaneous application of EEG monitoring and TMS allows measuring cerebral responses to stimulation. From here, the correlation between EEG response to TMS and individual cognitive profiles can be analyzed. Santarnecchi and Rossi [65] hypothesize that higher intelligence levels may result from greater integration in brain mechanisms. Santarnecchi et al. [67] showed that stimulation effects are related to individuals’ baseline abilities. Those with lower ability levels at baseline obtain more benefits from stimulation, whereas those with better ability levels at baseline are “*already optimized for the task at hand and therefore (they are) shielded against perturbation*”. The individual’s sensitivity to exogenous perturbations may be one critical feature of the brain, and, also, of general cognitive ability.

Individual differences in sensitivity to exogenous inputs can be related to the findings summarized, discussed and integrated here. We found greater gray matter changes in those individuals with smaller gains across the dual n-back training program. Furthermore, individuals showing large training gains achieved their high performance levels without modifying their brain structure. These ‘shielded’ individuals had higher fluid reasoning ability scores at baseline, and, therefore, we concluded that they had the cognitive power required for coping successfully with the challenging training. Finally, we found that training did have special impact on brain structures in those individuals showing lower fluid reasoning ability scores at baseline.

In conclusion, we strongly think it is time to switch our perspective regarding the most efficient way for achieving the goal of enhancing intelligence. We must move the focus for devoting full attention to the individual. Training programs must be properly personalized. Individual cognitive profiles must be specified before designing the intervention best fitted for enhancing the ability of interest. This reminds us the venerable aptitude × treatment interaction (ATI) approach [68,69]. One size fails to fit all. Achieving the demanding goal of enhancing intelligence will require one huge integrative and multidisciplinary effort, but it is greatly worthwhile [12].

## Figures and Tables

**Table 1:** Reliability (Cronbach’s alpha values) for all intelligence measures. RAPM = Raven Advanced Progressive Matrices, DAT-AR = abstract reasoning subtest from the Differential Aptitude Test, PMA-R = inductive reasoning subtest from the Primary Mental Abilities Battery, DAT-VR = verbal reasoning subtest from the DAT, DAT-NR = numerical reasoning subtest from the DAT, PMA-V = vocabulary subtest from the PMA. Gf = fluid intelligence, Gc = crystallized intelligence.

Intelligence Measures	Cronbach’s Alpha	Spearman–Brown Correction
Gf-RAPM	0.630	0.773
Gf-DAT-AR	0.675	0.806
Gf-PMA-R	0.765	0.867
Gc-DAT-VR	0.627	0.771
Gc-DAT-NR	0.675	0.808
Gc-PMA-V	0.835	0.910
